# Parental motivations and perceived barriers to participating in pediatric vaccine clinical trials: Findings from the OCV-S trial in Nepal

**DOI:** 10.1016/j.jvacx.2024.100585

**Published:** 2024-11-22

**Authors:** Ram Hari Chapagain, Santosh Adhikari, Kshitij Kunwar, Prabhat Thapa, Jessica Maharjan, Bishnu Rath Giri, Nisha Jyoti Shrestha, Anil Kumar Shrestha, Sanjeet Kumar Shrestha, Suresh Man Tamang, Deok Ryun Kim, Tarun Saluja, Anh Wartel, Julia Lynch, Katerina Rok Song

**Affiliations:** a OCV-S Study Site, Kanti Children’s Hospital, Kathmandu, Nepal; b National Academy of Medical Sciences (NAMS), Kathmandu, Nepal; c International Vaccine Institute (IVI), Korea

**Keywords:** Clinical Trials, Pediatric Vaccine Trials, Parental Motivation, Recruitment Barriers, Participant Retention, Clinical Trial Enrollment, Nepal, OCV-S trial

## Abstract

**Background:**

Pediatric vaccine clinical trials are crucial for evaluating and ensuring the safety and efficacy of new vaccines for children. However, in low-resource settings like Nepal, where clinical trials are relatively new, recruitment and retention of participants are challenging particularly due to diverse parental backgrounds, motivations and concerns. As such, parental motivations, perceived barriers, and experiences of participating in pediatric vaccine trial in Nepal, which hasn’t previously been explored, needs to be understood.

**Materials and Methods:**

We performed face-to-face exit interviews from April 6, 2022 to June 7, 2022, with parents whose children participated in the phase III clinical trial of the Oral Cholera Vaccine − Simplified (OCV-S) using a structured questionnaire on motivations for enrolling and barriers faced during the trial. Data were initially cleaned and encoded in Microsoft Excel before being analyzed with R version 4.3.1. Descriptive statistics were used to summarize demographic and other participants’ characteristics. Bivariate and multivariate analyses, using odds ratios and their 95% confidence intervals, applying a significance level of 0.05 was used to analyze relationship between participant characteristics and trial experiences. Additionally, thematic analysis was performed on responses to open-ended questions.

**Results:**

A total of 258 parents responded, out of which 252 (97.7 %) were first-time clinical trial participants with median age of 32 years. A majority, 196 (76.0 %), had a positive initial attitude towards the trial, and 204 (79.1 %) reported a positive overall experience. The primary motivations for participation included the potential health benefits for their children (56.2 %, n = 145). Despite 69 (26.7 %) participants receiving discouraging information from others, only 4 (5.8 %) were influenced by it. Most participants (n = 219, 84.9 %), felt that the trial had met their expectations. Challenges faced included loss of time (n = 30, 11.6 %) and missed school for children (n = 22, 8.5 %). Nonetheless, 179 participants (69.4 %) expressed a willingness to enroll their children in future trials. Participants who received specific details about the study before visiting the study site were significantly more likely to report positive experience, with an adjusted odds ratio of 1.97 (95 % CI: 1.03 – 3.72).

**Conclusion:**

Majority of parents were supportive of their children’s participation in the OCV-S trial, motivated largely by anticipated health benefits for their child. Key barriers identified included logistical issues, costs (both financial and opportunity costs), misinformation, and concerns about trial procedures and potential side effects. Focus on reducing logistical and participation-related burdens, catering of trial-specific information, enhancing the clarity of the informed consent process, addressing safety concerns proactively and implementing continuous follow-up can help improve participation rates and retention in future trials. The high level of enthusiasm for clinical trials among parents, despite these barriers, highlights the promising potential for future research endeavors in Nepal.

## Background

The need for clinical trials in children has been increasingly recognized by the scientific community. Clinical trials in the pediatric population are more challenging than those in adults [1]. Parents are expected to act in the best interest of their child and hence have been entrusted with the responsibility of providing permission or consent for enrolling their children in research studies as well as supporting the study protocol [2], [3]. Informed consent from parents as well as informed assent needs to be secured from children above a certain age for the enrollment of the child in the study. Ensuring participant retention throughout the trial is essential for meeting study requirements and maintaining protocol adherence. Because the recruitment process is often a long and laborious exercise, participant retention is important, as dropouts can lead to increased trial costs, longer trial duration, less statistical power for the study and the validity of the results, and low staff morale [2]. Any dropouts or lost-to-follow-up is a loss to the study because those participants may not be eligible for inclusion in the final analysis set. High recruitment and retention rates in randomized controlled trials are essential to ensure validity and broad generalizability [4].

Participant recruitment and retention are two significant challenges in conducting clinical trials and contribute largely to the delays in development of potential treatments [5]. Each study aspect, from the initial explanation of the study to the end of the study, can affect the willingness of parents to consent to the proposed study and future studies [2].

Effective study design must ensure compliance with inclusion criteria and address factors influencing dropout rates. Understanding barriers and motivators for completing the study is crucial for developing strategies to enhance trial quality.

The barriers can vary between participants by their backgrounds. Support during initial decision making, throughout the trial phase, parental race, parental education level, perceived advantages and disadvantages of clinical trials, altruism were predictors of parental trust in medical researchers for child and adolescent participation in clinical trials [6], [7], [8], [9], [10], [11], [12].

The parental motivating factors were desire to help others and to contribute to medical research, better ways to help their own children, free medication, low economic condition and socially disadvantaged and emotionally vulnerability [13], [14].

Despite the facts some study showed access to free medication was more important in families with lower incomes than in families with higher incomes and high-income families are more chance to decline the consenting process [15], [16]. If they consented, chances of retention are higher than that of lower income group [17]. Which means economic characteristics seem to affect parents’ consent to pediatric clinical trials.

There are clinical trials going on in Nepal. OCV-S phase III trial is one of the pivotal trials for testing the immunogenicity of newly formulated oral cholera vaccine in the cholera endemic countries and it has been conducted during the late phase of second wave of the COVID-19 pandemic. The importance of the clinical trials has been felt by regulatory authority so that there are different guidelines and SOP prepared for the conduction of clinical trials. Some successful trials have been completed in COVID era like typhoid vaccine, oral cholera simplified vaccine [18], [19]. There are scarce publication regarding the motivation and barriers of parents participating in clinical trials while searching the portals like PubMed, Chrocane, Google Scholar and others.

This study aims to address a significant gap in the literature by examining the factors that influence parental decisions regarding their child's participation in pediatric vaccine clinical trials. Specifically, it investigates the key motivations and barriers faced by parents in the context of a pediatric vaccine trial conducted in Nepal. The research aims to understand how these factors impact parental decision-making and their overall experience with the trial, offering insights into the unique challenges and considerations in this low-resource setting.

## Materials and methods

### Study setting

This is a sub-study of the study entitled “A Phase III, Multicenter, Observer-Blinded, Randomized, Active Controlled Trial to Evaluate Immune Non-Inferiority, Safety and Lot-to-Lot Consistency of Oral Cholera Vaccine-Simplified Compared to Shanchol™ in 1 to 40 years old Healthy Nepalese Participants” [20]. The approval has been taken from Nepal Health Research Council (NHRC), registration no. 292/2021, Department of Drug administration (DDA), Council of Ministers. Out of approximately 625 parents visiting the trial site for the final on-site visit of the study at Kanti Children’s Hospital, 258 participated in the exit interview which was conducted from April 6, 2022, to June 7, 2022. Participants were briefed about the study and how their privacy and confidentiality would be maintained. They were also made aware that their participation was voluntary, that they could freely answer the questions without having to necessarily provide a reason for their response and could withdraw their participation at any time without citing any reason or facing any consequences.

### Study design

Parents or legal guardians of participants enrolled in the OCV-S study who attended the final on-site follow-up visit were included in this study. Convenience sampling was used for participant recruitment, as the study involved exit interviews with individuals available at the time. The inclusion criteria were: (1) Aged 18 years or older, (2) Nepali-speaking, and (3) A legal guardian or primary caregiver of an OCV-S study participant who consented to participate in this sub-study. Interviews continued until the required sample size was reached. See Table 1, Table 2.

**Table 1:** Demographic charac**teristics of the participants (N = 258)**.

Characteristics	n (%)
Relationship to the child	
Mother	200 (77.5)
Father	58 (22.5)
Age	
Median age = 32, IQR = 8 years	
<30 years	92 (35.6)
30–44 years	150 (58.1)
45–59 years	16 (6.2)
Ethnicity	
Brahaman/Chhetri	80 (31.0)
Janajati	131 (50.8)
Dalit	44 (17.0)
Tarai/Madhesi	3 (1.2)
Permanent residence	
Inside Kathmandu valley	60 (23.3)
Outside Kathmandu valley	198 (76.7)
Highest Level of Education Completed	
Literate	150 (58.1)
High school completed	89 (34.5)
College/university completed	19 (7.4)
Chief occupation	
Agriculture	11 (4.2)
Employment	82 (31.8)
Self-Employed	58 (22.5)
Home-maker	107 (41.4.)
Family type	
Nuclear	220 (85.3)
Extended	38 (14.7)

**Table 2:** Characteristics of the participants related to the trial participation (N = 258).

Characteristics	n (%)
Prior Acquaintance with Other Study Participants	
Yes	162 (62.8)
No	96 (37.2)
Source of Information Regarding the Clinical Trial	
Health personnel	119 (46.1)
Non-health personnel	139 (53.9)
Nature of Initial Information Received About the Clinical Trial	
General information	185 (71.7)
Specific information	71 (27.5)
Don’t know	2 (0.8)
Initial Attitude Towards the Clinical Trial Upon Awareness	
Positive	196 (76.0)
Negative	25 (9.7)
Neutral/Curious	37 (14.3)
Previous Participation of Child in any Clinical Trials	
Yes	29 (11.2)
No	229 (88.8)
Previous Participation of Parent in Clinical Trials	
Yes	6 (2.3)
No	252 (97.7)
Overall Experience with the Current Clinical Trial	
Extremely positive	11 (4.3)
Positive	193 (74.8)
Neutral	54 (21.0)
Negative	0 (0 %)
Extremely negative	0 (0 %)
Sources of Assistance for Decision-Making Regarding Child’s Participation	
No assistance	36 (14.0)
Only Family members	160 (62.0)
Family members and neighbors/relatives	43 (16.7)
Family members and family doctor	19 (7.4)
Types of Feedback Received from Others About Participation	
None	146 (56.6)
Both encouraging and discouraging type	75 (29.0)
Only Encouraging-type	22 (8.5)
Only Discouraging-type	14 (5.4)
Don’t know	1 (0.4)
Presence of Questions During the Consent Process	
Yes	108 (41.9)
No	121 (46.9)
Don’t know	29 (11.2)
Types of Questions Asked During the Consent Process (Multiple Responses)	
Child safety	102 (39.5)
Study procedure	26 (10.1)
Benefits of participating	21 (8.1)
Other questions	21 (8.1)
Motivations for Participation in the Clinical Trial	
Reputation of the Study Site	7 (2.7)
Previous Trial Experience	2 (0.8)
Personal Belief in Vaccines	9 (3.5)
Persuaded by Other Participants	36 (14.0)
Persuaded by Health Workers	22 (8.5)
Desire for Improved Health of Child	145 (56.2)

Desire to Prevent Cholera	35 (13.6)
Don’t know	2 (0.8)
Consideration of Discontinuing the Trial	
Yes	21 (8.1)
No	237 (91.9)
Reasons for Considering Discontinuation of the Study	
Fear of possible side-effects of the study vaccine	9 (42.8)
Lack of time for participation	6 (28.7)
Difficult study procedure	5 (23.8)
Negative information received about the study	1 (4.7)
Has Interest in Future Clinical Trials for the Child	
Yes	179 (69.4)
No	8 (3.1)
Unsure	71 (27.5)
Exposure to Discouraging Information During the Trial	
Yes	69 (26.7)
No	189 (73.3)
Types of Discouraging Information Heard	
Taking part in clinical trials is harmful to children	41 (15.9)
Clinical trials mean selling the blood of your children	27 (10.5)
Clinical trials are done only among poor population	1 (0.4)
Influence/Effect of Discouraging Information on Participation Decision	
Yes	4 (5.8)
No	65 (94.2)
Alignment of Trial Experience with Expectations	

As Expected	219 (84.9)
Not as Expected	21 (8.1)
Don’t know	18 (7.0)
Likelihood of Recommending Participation in Similar Studies	
Yes	206 (79.9)
No	17 (6.6)
Unsure	35 (13.6)
Perceived Benefits from Participation in the Clinical Trial	
Improved health and immunity of child	123 (47.7)
No major adverse event	22 (8.5)
Free Health Care for Child	15 (5.8)
Increased Knowledge About Clinical Trials	1 (0.4)
No Specific Benefits	92 (35.7)
Don’t know	5 (1.9)
Perceived Losses Due to Participation in the Clinical Trial	
Loss of time/duty hour of parent	30 (11.6)
Child had to miss school	22 (8.5)
Impaired Child Health	20 (7.6)
Increased Fear of Side Effects	2 (0.8)
No Perceived Losses	184 (71.3)

### Data collection

Following the informed consent, participants were interviewed face-to-face using a structured questionnaire from April 6, 2022, to June 7, 2022. The development of the questionnaire involved several meetings with clinical trial investigators and site staff to draft, review, and finalize the questions, ensuring the validity and relevance of the content. The questionnaire covered a range of topics including demographic characteristics (age, sex, residence, ethnicity, educational status, chief occupation, family type), information about the trial prior to participation, sources of information about the trial, initial impressions of the vaccine, and prior experience with clinical trials. Additionally, the survey explored motivational factors (such as personal beliefs and child’s health), any thoughts of withdrawing from the trial, and intentions to participate in future trials. All study variables except age of participants (which is a discrete numerical variable) were categorical variables. Feedback on the trial experience, including perceived benefits (such as improved health status and free healthcare), perceived losses, and aspects participants liked or disliked, was also collected. All responses were recorded by the interviewer during the exit interviews.

### Sample size calculation

The sample size was determined based on the literature indicating that 81.1 % of participants are motivated by the hope of protection against COVID-19 [21].

n=z^2^*p*(1−p)/e^2^

where:

*n is the sample size,*

*z is the z-score associated with 95 % confidence (1.96),*

p is the proportion of the motivating factor (0.811),

*e is the margin of error, expressed as a decimal = 0.05,*

Non-response rate = 10 %.

Total sample size (with adjustment for non-response) = 258.

### Data analysis

Descriptive statistics were used to summarize demographic and other participants’ characteristics. Categorical variables were reported as frequencies and percentages.

Bivariate analyses were conducted to assess the relationships between variables, while multivariate analyses, utilizing logistic regression, calculated odds ratios (OR) and adjusted odds ratios (AOR) with 95 % confidence intervals (CI) and a significance level of 0.05 to analyze participant characteristics across three key outcomes: consideration of trial discontinuation, clinical trial experience, and likelihood to recommend future clinical trials.

**Consideration of Trial Discontinuation:** Both univariate and multivariate logistic regression models were employed. The univariate analysis examined individual characteristics—including age, ethnicity, highest level of education, and source of information—to determine their associations with the outcome of considering trial discontinuation. The multivariate model simultaneously included all variables to adjust for potential confounding factors, thereby enabling the assessment of the independent effects of each characteristic on the likelihood of participants contemplating discontinuation.

**Clinical Trial Experience:** A similar analytical framework was utilized to evaluate clinical trial experience. Univariate analyses identified individual associations, while multivariate logistic regression controlled for variables such as age, ethnicity, type of information received, and family type. This methodology was designed to elucidate how these factors influenced participants' experiences within the trial context.

**Likelihood to Recommend Future Clinical Trials:** For the evaluation of factors influencing the likelihood of recommending future participation in clinical trials, both univariate and multivariate logistic regression models were employed. The multivariate model adjusted for age, ethnicity, educational level, family type, source of information, and the presence of questions during the consent process.

Additionally, thematic analysis was employed to interpret qualitative data from open-ended questions.

## Results

### Demographic characteristics of participants

The median age of the participants was 32 years, with 150 (58.1 %) falling within the 30 to 44-year age range. The cohort was predominantly composed of mothers (77.5 %, n = 200), and a significant portion (76.7 %, n = 198) resided outside the Kathmandu valley. Ethnically, 80 (31.0 %) were Brahman/Chhetri, 131 (50.8 %) Janajati, 44 (17.0 %) Dalit, and 3 (1.2 %) Tarai/Madhesi. All participants were literate, with no illiterate individuals included. Employment was common, with 199 (77 %) engaged in various occupations, predominantly self-employed or business owners. Additionally, 220 (85.3 %) participants came from nuclear families.

### Characteristics related to trial participation

About 162 (62.8 %) of participants were familiar with other trial participants, receiving information from both medical (46.1 %, n = 119) and non-medical sources (53.9 %, n = 139). Most participants (71.7 %, n = 185) received general information about the trial, while 71 (27.5 %) received specific details such as study procedures and potential adverse events. Initial attitudes towards the trial were largely positive, with 196 (76.0 %) viewing it favorably upon first learning about it. Notably, 229 (88.8 %) were new to the concept of clinical trials.

During the trial, 204 (79.1 %) reported a positive overall experience. Before enrolling, 160 (62.0 %) consulted family members, and 112 (43.4 %) received feedback from others, with 146 (56.6 %) receiving no comments, 75 (29.0 %) receiving both encouraging and discouraging feedback, and 14 (5.4 %) receiving only discouraging comments.

In the consent process, 108 (41.9 %) had questions, predominantly about child safety (39.5 %, n = 102). The most significant motivation for participation was the anticipated health benefits for the child (56.2 %, n = 145), with 123 (47.7 %) perceiving increased immunity and improved health. The trial met the expectations of 219 (84.9 %) participants. Further, 206 (80 %) reported that they would recommend others for clinical trial participation in future. Participants with at least high-school education were statistically more likely to recommend future trials compared to those with less that high-school education.

The study also showed that (21 (8.1 %) considered discontinuation, primarily due to fear of side effects (42.8 %, n = 9) and lack of time (28.7 %, n = 6). Despite 69 (26.7 %) being exposed to discouraging information, only 4 (5.8 %) were influenced by it. Discouraging information included beliefs that clinical trials are harmful (15.9 %, n = 41) or involve selling blood (10.5 %, n = 27).

The statistical analysis of participant characteristics and their consideration of trial discontinuation showed no significant associations across the variables examined. Specifically, age, ethnicity, and highest level of education did not significantly influence the likelihood of participants considering discontinuation. Additionally, the source of information about the trial—whether obtained from health workers or non-health workers—did not show a statistically significant impact on the decision to discontinue. Thus, none of the variables studied had a significant effect on participants' consideration of discontinuing the trial. Detailed results of the analysis are presented in Table 3.

**Table 3:** Statistical Analysis of Participant Characteristics and Their Consideration of Trial Discontinuation.

Characteristics	Had ever thought of discontinuing the trial		p-value	OR (95 % CI)	AOR (95 % CI)	p-value
	Yes n (%)	No n (%)				
Age of participant						
< 32 years	11 (9.4)	106 (90.6)	0.49	1.36 (0.56–3.32)	1.34 (0.54–3.39)	0.53
≥ 32 years	10 (7.1)	131 (92.9)				

Ethnicity						
Brahmin/Chhetri	7 (8.8)	73 (91.2)	0.81	1.12 (0.44 – 2.90)	0.76 (0.25–2.09)	0.59
Others	14 (7.9)	164 (92.1)				

Highest level of education completed						
High school completed	12 (11.1)	96 (88.9)	0.14	0.51 (0.21 – 1.26)	0.52 (0.19 – 1.37)	0.19
High school not completed	9 (6.0)	141 (94.0)				

Source of information about the trial						
Health worker	13 (10.9 %)	106 (89.1 %)	0.13	−.) 2.01 (0.80––5.03)	2.01 (0.78 – 5.42)	0.15
Non-health worker	8 (5.8 %)	131 (94.2 %)				

The analysis showed that participants who received specific information about the trial reported a significantly more positive experience compared to those who received general information (AOR 1.97, 95 % CI: 1.03–3.72; p = 0.0373). No significant differences were observed in trial experience based on age, ethnicity, or family type. These findings are detailed in Table 4.

**Table 4:** Statistical Analysis of Participant Characteristics and Their Clinical Trial Experience.

Characteristics	Clinical trial experience		p-value	OR (95 % CI)	AOR (CI)	p-value
	Neutral n (%)	Positive n (%)				
Age of participant						
< 32 years	25 (21.4)	92 (78.6)	0.87	1.05 (0.57 – 1.92)	0.93 (0.5–1.75)	0.824
≥ 32 years	29 (20.6)	112 (79.4)				

Ethnicity						
Brahmin/Chhetri	13 (16.2)	67 (83.8)	0.22	1.54 (0.77–3.07)	1.52 (0.77–3.14)	0.242
Others	41 (23.0)	137 (77.0)				

Nature of first information received about clinical trial						
General	33 (17.8)	152 (82.2)	0.035	1.96 (1.04 – 3.69)	1.97 (1.03–3.72)	0.0373
Specific	21 (28.8)	50 (71.2)				

Type of family						
Nuclear	43 (19.5)	177 (80.5)	0.19	1.67 (0.77 – 3.65)	1.71 (0.74–3.77)	0.191
Extended	11 (28.9)	27 (71.1)				

The analysis indicated that ethnicity was a significant factor influencing the likelihood to recommend future clinical trials. Participants from the Brahmin/Chhetri group were more likely to recommend participation compared to those from other ethnicities (AOR 2.00, 95 % CI: 0.90–4.84; p = 0.10), though this finding approached significance. Additionally, participants who had questions during the consent process were more likely to recommend future clinical trials (AOR 1.93, 95 % CI: 1.00–3.85; p = 0.05). Other variables, including age, educational level, family type, and source of information, did not show significant associations with the likelihood to recommend participation. Detailed results are presented in Table 5.

**Table 5:** Test of Statistical Significance for Participant Characteristics and Likelihood to Recommend Future Clinical Trials.

**Characteristics**	**Suggests others to participate in clinical trials**		**p-value**	**OR (95 % CI)**	**AOR (CI)**	**p-value**
	**Yes** **n (%)**	**No/Unsure** **n (%)**				
Age of participant						
< 32 years	104 (78.8)	28 (21.2)	0.66	0.87 (0.47 – 1.60)	0.88 (0.46–1.68)	0.70
≥ 32 years	102 (81.0)	24 (19.0)				

Ethnicity						
Brahmin/Chhetri	71 (88.8)	9 (11.2)	0.02	2.51 (1.16 – 5.45)	2 (0.9–4.84)	0.10
Others	135 (75.8)	43 (24.2)				

Educational level						
Literate	113 (75.3)	37 (24.7)	0.03	0.49 (0.25 – 0.95)	0.66 (0.32–1.33)	0.25
High school completed or higher	93 (86.1)	15 (13.9)				
Type of family						
Nuclear	175 (79.5)	45 (20.5)	0.77	0.88 (0.36 – 2.12)	0.99 (0.36–2.45)	0.97
Extended	31 (81.6)	7 (18.4)				

Source of information						
Health worker	101 (84.9)	34 (24.5)	0.06	1.81 (0.96 – 3.42)	1.58 (0.8–3.19)	0.19
Non-health worker	105 (75.5)	18 (15.1)				

Presence of Questions During the Consent Process						
Yes	16	92	0.07	1.82 (0.94 – 3.48)	1.93 (1.00–3.85)	0.05
No/Don’t know	36	114				

## Discussion

Despite less population, due to increased scientific awareness, growing capacity and diverse ethnicity and population, Nepal is increasingly being recognized as a potential site for health research along with clinical trials. Enrollment and sustained participation are undoubtedly the most important phases of a clinical trial [2], [7], [22], [23]. According to the NIH, about 80 % of the clinical trials in the US missed their timelines due to issues on patient’s recruitment and enrollment. With 11 % of clinical research sites failing to enroll even a single participant, and 37 % of sites under-enrolling, recruitment strategies must be considered à priori and addressed throughout the duration of the study [24]. This setback increases research costs, and can delay the approval of the therapeutic intervention under investigation [2], [5] So it is important to identify the reasons driving participation in clinical studies in order to ensure that the clinical trial does not suffer from high dropouts, and the results emerging from the trial have robust validity [25].

Retention in a clinical trial is defined as the strategy and tactics designed to keep participants enrolled in clinical trials, from discontinuing participation and dropping out [26]. A successful participant retention strategy involves treating the participant with respect [12], being considerate of the participant's time, identifying and overcoming barriers to retention in a timely manner [27], [28].

The present study describes the motivating and demotivating factors experienced by the parents who completed the OCV-S (Oral Cholera Vaccine-Simplified) trial at Kanti children’s Hospital site, Nepal. Sixty-three percent (n = 162) parents knew other participants before their child participated in this trial, meaning that most of the parents came with some recommendations. More than half (53.9 %, n = 139) received information from people who were from a non– medical background. Other studies have shown that more children participated in trials if they are told to participate by their family doctors and those who were reluctant to participate were ready to participate with fear if they were explained by doctor [4], [12], [21]. This indicates that parents might trust all people from both medical and non-medical background for information related to clinical trials. Majority (76.0 %, n = 196) of the parents had a positive attitude initially about the trial. However, 62 (24 %) either had a negative attitude or were indecisive initially but later on they decided to join and completed the participation in the trial. These potential participants should be well explained to positively shape their attitude to the trial and to mention their coheirship till the trial period. This shows the importance of pre-consenting information and informed consent procedure.

In our study, almost all (97.7 %, n = 252) were first time participants in a clinical trial which means curious participants may have enrolled and continued the trial. This has also been observed by other studies. A study conducted by Finn R et al. observed that 71 % of participants who were aware of clinical trials rejected to participate [29]. This might be the cause that people who have never participated in a trial before show more interest in enrolling in clinical trials with decline in interest in future trials.

Among the parents participating in OCV-S trial, almost eight out of ten (79.1 %, n = 204) had an extremely nice or nice experience and the rest reported having a neutral experience during the study. The decision-making process of participating in a clinical trial is complex and influenced by many factors like recruitment practice, perceived benefit [7], [8], personnel benefit [10], [11], health benefit of child, altruism, trust in research and related researcher [2], [9]. Only 36 (14.0 %) took the decision of participating their child in OCV-S study by themselves but majority (62.0 %, n = 160) took help from family. Likewise, 146 (56.6 %, n = 146) had not received comments for others for participating their child to OCV-S trial and one third (29.0 %, n = 75) received both encouraging and discouraging. This is important on continuation of the follow up on clinical trial.

Informed consent is essential as well as an important component of the clinical trial which gives detailed understanding and motivation for participation in trial and helps to complete the follow up without dropout. In our study, we observed 108 (41.9 %) had questions during consenting. Majority (39.6 %, n = 102) of questions were related to child’s safety followed questions about by study procedure (10.1 %, n = 26). The importance of consent was also highlighted by other studies [2], [14], [30]. In contrast to ours, a qualitative study conducted by Woolfall, K et al found that while parents had many questions and concerns about trial participation which influenced their decision-making, they rarely voiced these during discussions about the trials with practitioners and they recommend that those involved in the recruitment of children to clinical trials need to be aware of parents' priorities and the sorts of misunderstandings that can arise with parents. Providing trial information that is tailored to what parents consider important in deciding about a clinical trial may improve recruitment practice and ultimately benefit evidence-based pediatric medicine [8].

Motivation is important for successful completion of clinical trials and decreasing the dropout. Some studies showed that health benefits for children, altruism, trust in research, and relation to researchers were the most common motivating factors of parents and personal health benefit, altruism and increasing comfort were for children [9], [31], [32].

Present study showed that the main motivating factors for participating in OCV-S was the possibility of improvement of health in 145 (56.2 %) participants followed by being convinced by other participants and prevention from the Cholera (14.0 % and 13.6 % respectively).

The other factors were that the trial was carried out in a renowned hospital, personal faith in vaccines and conviction by other health workers. Our study showed that the 21 (8.1 %) parents had thoughts about discontinuing the trial during the trial period but they kept continuing and came for follow-up till the end of the trial.

“Taking part in clinical trials is harmful”, “Clinical trials take blood samples to other places for commercial purposes” and “Clinical trials are done only among very poor population” are some of the discouraging information heard by parents [33]. Only 69 (26.7 %) heard such discouraging information during the trial. Though they heard the discouraging information, only 4 (5.8 %) were really influenced by the discouraging information. It was observed that 219 (84.9 %) respondents expressed that the trial went as per their expectation after consenting to enroll their child in the trial. These are similar to other studies which stated fear of risks, distrust in research, logistical aspects and disruption of daily life were mentioned most by parents as discouraging factors. Burden and disruption of daily life, feeling like a “guinea pig” and fear of risks were most mentioned as discouraging factors by children [9].

Participants largely valued the clinical trial for its positive impact on their child’s health and its effectiveness in preventing cholera. They appreciated the convenience of the trial, such as the oral vaccine administration and minimal disruption to daily routines. Support from trial staff, including effective counseling and attentive care, was also highly rated. Overall, feedback indicated a high level of satisfaction with the trial’s outcomes and process.

Participants expressed concerns about procedural and administrative aspects, particularly the blood collection process and stringent eligibility criteria. Some reported negative health effects on their children and dissatisfaction with the discontinuation of care post-trial. Issues related to loss of time and insufficient information were also noted. However, most participants did not have specific complaints, suggesting these issues were relatively uncommon.

Participants suggested several improvements to enhance the trial experience. Key recommendations included streamlining site operations, such as optimizing blood collection and site access, and reducing waiting times. Enhancing procedural efficiency and minimizing paperwork were also recommended.

The perceived benefits are the major factors for taking part and continuing the trial [8], [11]. This present study showed that 123 (47.7 %) parents perceived health and immunity of the child as the benefit from participation and more than one third 92 (35.7 %) took part and completed the trial without having perceived benefit from participation.

Most of the participants reported having lost time due to trial enrollment and follow-ups and 22 (8.5 %) were concerned about their children missing school. This highlights the importance of opening the trial site for extra hours in case of pediatric recruitment. Quite a negligible 2 (0.8 %) number of parents feared vaccine side effects. This shows the importance of informed consent process and pre-explanation of the study product and procedure related information. This also highlights the areas which researcher should emphasize throughout out the study to decrease the dropout rate in clinical trial.

It is interesting to note that only 9 (3.1 %) said they do not want to take part in similar future clinical trials while 179 (69.4 %) parents wanted to enroll their child in future studies. This shows the tremendous potential of smooth vaccine related clinical trials in Nepal in the future.

It was observed that the extended family membered are more willing to take participants which might be the decision making facilitated by the family members.

It was also found that the participant who had at least completed high-school education were statistically significantly more likely to suggest other people to take part in clinical trials in the future compared to those who had not completed a high-school education. This hypothesizes that people with higher level of education are comparatively more positive about participation in clinical trials. But except the formal education a study conducted in 2023 shown that the power of education is not sufficient to overcome barriers to clinical trial participation for underrepresented communities [34].

The multivariate analysis revealed that participant characteristics such as age, ethnicity, and education level did not significantly influence their likelihood to recommend future clinical trials or their overall experience with the current trial. However, participants who received specific information about the trial reported a significantly more positive experience compared to those who received general information.

## Conclusion

The study highlights that while participants generally had a positive experience with the OCV-S clinical trial, the provision of specific information significantly improved their satisfaction. Participants were primarily motivated by concerns for their child’s health and disease prevention. Additionally, encouragement from friends, family, and neighbors, as well as recommendations from health workers, notably influenced their decision to participate.

### Barriers and motivations

Barriers: The primary barriers to continuing the OCV-S trial were fears of possible side effects (42.8 %), lack of time (28.7 %), and study procedures perceived as difficult (23.8 %). Despite these concerns, the majority of participants (91.9 %) did not consider discontinuing the trial.

Motivations: The key motivation for participation to OCV-S was the desire for the improved health of the child (56.2 %), followed by the intention to prevent cholera (13.6 %). Most participants also believed in the potential benefits of the trial, such as improved immunity for the child (47.7 %) and free health care (5.8 %). Participants were also motivated by encouragement from friends, family, and neighbors, which played a significant role in their decision to participate.

Experiences: Overall, the trial experience was positive for most participants, with 74.8 % reporting a positive or extremely positive experience. Only 8.1 % felt neutral about the trial experience, and no participants reported an extremely negative experience. Participants who received specific information about the trial reported a significantly more positive experience (AOR = 1.97, 95 % CI: 1.03–3.72, p = 0.0373) compared to those who received general information.

### Recommendations

To enhance future trials, it is important to provide detailed and specific information about the trial to participants to address concerns comprehensively. Reducing barriers such as time constraints and enhancing support systems will improve participant experiences. Emphasizing the benefits of participation and effectively addressing any negative perceptions will also promote greater engagement and satisfaction. Additionally, leveraging recommendations from health workers and incorporating support from friends and family can further influence positive participation outcomes.

### Strengths and limitations

This study is one of the first to explore parental motivations and barriers specific to pediatric vaccine clinical trials within a low-resource setting like Nepal and addresses a significant gap in the existing literature. The exit interview format provided participants the opportunity to respond to questions about their recent trial experiences, minimizing recall bias and ensuring accuracy.

The study has several limitations too. We found that a significantly large number of the children were brought for the vaccination by mothers (77.5 %, n = 200) so the majority of the participants were female in our study. Mothers were over-represented in the sample as fathers were either not present in recorded trial discussions or did not participate in interviews as a result, potential gender differences in the understanding of parents could not be explored. The motivation and barriers were explored from parents who completed the trial successfully so the view of the parents who left the trial before completing the study could not be explored. = The study also relies on self-reported data, which could introduce response bias.

## Authors Contributions

RHC, SA, KK, JM contributed to the study conceptualization, design and analysis. JM, KK, SMT, AKS, SS, NJS, and PT were involved in the study implementation. RHC, SA, and KK drafted the manuscript. JL, DRK, TS, AW, BRG, BKG, KRS, SA, JM, PT, SMT, NJS, AKS, SKS made the revisions to the final version of the manuscript. All authors had full access over the manuscript and the corresponding author had final responsibility for the decision to submit for the publication.

## CRediT authorship contribution statement

**Ram Hari Chapagain:** Writing – review & editing, Writing – original draft, Formal analysis, Conceptualization. **Santosh Adhikari:** Writing – review & editing, Writing – original draft, Supervision, Project administration, Methodology, Formal analysis, Conceptualization. **Kshitij Kunwar:** Writing – review & editing, Writing – original draft, Conceptualization. **Prabhat Thapa:** Writing – review & editing, Supervision, Project administration, Data curation, Conceptualization. **Jessica Maharjan:** Writing – review & editing, Writing – original draft, Methodology. **Bishnu Rath Giri:** Writing – review & editing, Writing – original draft. **Nisha Jyoti Shrestha:** Writing – review & editing, Writing – original draft. **Anil Kumar Shrestha:** Writing – review & editing, Writing – original draft. **Sanjeet Kumar Shrestha:** . **Suresh Man Tamang:** Writing – review & editing, Writing – original draft. **Deok Ryun Kim:** Writing – review & editing, Formal analysis, Data curation. **Tarun Saluja:** Writing – review & editing, Supervision, Methodology. **Anh Wartel:** Writing – review & editing, Methodology, Conceptualization. **Julia Lynch:** Writing – review & editing, Funding acquisition. **Katerina Rok Song:** Writing – review & editing, Writing – original draft, Funding acquisition, Data acquisition, Conceptualization.

## Declaration of competing interest

The authors declare the following financial interests/personal relationships which may be considered as potential competing interests: Ram Hari Chapagain reports article publishing charges was provided by Bill & Melinda Gates Foundation.

## Data Availability

Data will be made available on request.
